# Effectiveness of a Quit Vaping Text Message Program in Promoting Abstinence Among Young Adult E-Cigarette Users: Protocol for a Randomized Controlled Trial

**DOI:** 10.2196/18327

**Published:** 2020-05-01

**Authors:** Amanda L Graham, Megan A Jacobs, Michael S Amato, Sarah Cha, Mia M Bottcher, George D Papandonatos

**Affiliations:** 1 Innovations Center Truth Initiative Washington, DC United States; 2 Department of Medicine Mayo Clinic College of Medicine and Science Rochester, MN United States; 3 Department of Oncology Georgetown University Medical Center Washington, DC United States; 4 Center for Statistical Sciences Brown University Providence, RI United States

**Keywords:** e-cigarettes, tobacco cessation, young adults, text messaging, telehealth

## Abstract

**Background:**

Millions of young adults currently vape electronic cigarettes (e-cigarettes), yet little research on vaping cessation interventions exists. Text messaging is a promising, scalable intervention strategy for delivering vaping cessation treatment.

**Objective:**

This study evaluates the effectiveness of a text message quit vaping program (*This is Quitting*) in promoting abstinence from e-cigarettes among young adults; examines changes in self-efficacy, perceived social norms, and social support for quitting as hypothesized mediators of effectiveness; and examines if treatment effectiveness is moderated by gender, race, ethnicity, or sexual minority status.

**Methods:**

Overall, 2600 young adult (aged 18-24 years) e-cigarette users in the United States will be recruited via web advertisements to participate in the study. Participants will be randomized to *This is Quitting* or an assessment-only control condition. The primary outcome measure is 30-day vaping abstinence at 7 months post enrollment.

**Results:**

Study recruitment began on December 18, 2019, and is projected to be completed by spring 2020. The final 7-month follow-up is anticipated to be completed by fall/winter 2020. Because this is the first-ever evaluation of a quit vaping program, we were unable to draw on existing literature to determine the appropriate sample size. Therefore, we examined abstinence rates among an initial pilot sample of 269 participants (*This is Quitting*: n=148 and control: n=121) who completed the 1-month follow-up to determine the final sample size. The 1-month response rate was 79.2% (213/269), with no difference between arms. Using intention-to-treat analyses that counted nonresponders as still vaping, 30-day abstinence rates were 16.2% (24/148) among those randomized to *This is Quitting* and 8.3% (10/121) among those randomized to control. A treatment difference of 16% vs 8% is detectable with 80% power at 2-sided alpha=.05 with 260/group (520 total). To detect treatment differences of this magnitude in a 20% subsample (eg, Hispanic or sexual minority young adult e-cigarette users), we will enroll 1300/group (2600 total).

**Conclusions:**

The scientific, clinical, and public health communities are desperate for cessation resources to address vaping among young people. This study is the first-ever comparative effectiveness trial of an intervention to help young people quit vaping. It focuses on evaluating the effectiveness of a theory-grounded, empirically informed text message intervention among young adults. The study is fully powered to examine potentially important subgroup differences among young people who are more vulnerable to e-cigarette use. Although potentially more challenging from a research ethics and pragmatic standpoint, evaluating quit vaping intervention approaches in teens is an important area for future research. Data from this trial will establish a benchmark of effectiveness for other vaping cessation programs and begin to create a body of evidence focused on how best to help young people break free from e-cigarettes.

**Trial Registration:**

ClinicalTrials.gov NCT04251273; https://clinicaltrials.gov/ct2/show/NCT04251273

**International Registered Report Identifier (IRRID):**

DERR1-10.2196/18327

## Introduction

### Background

After decades of declining smoking rates, young people are returning to tobacco by vaping electronic cigarettes (e-cigarettes). E-cigarettes are currently the most heavily used tobacco product by youth and young adults [1]. According to the 2019 National Youth Tobacco Survey, 27.5% of high school students and 10.5% of middle school students reported using an e-cigarette within the past 30 days [2]. Data from the Centers for Disease Control and Prevention’s National Health Interview Survey showed an increase in e-cigarette use among young adults aged 18 to 24 years, from 5.2% in 2014 to 7.6% in 2018 [3]. Young adults are more likely to use e-cigarettes compared with adults older than 25 years. Recent data also suggest that members of some demographic groups, including men and young adults who identify as Hispanic or as a sexual minority, have a higher prevalence of e-cigarette use than others [4].

E-cigarette use among young adults is associated with future initiation of combustible tobacco use [5,6] and with increased odds of alcohol and marijuana use [7]. However, even if young e-cigarette users do not progress to other products or substances, early exposure to nicotine puts them at risk for a lifetime of addiction as well as largely unknown health risks of long-term e-cigarette use. The majority of e-cigarettes contain nicotine, and the concentrations available in popular products have increased over the past decade [8]. Nicotine has known health effects on brain development occurring into the mid-20s [9]. Specific risks include nicotine addiction, mood disorders, permanent lowering of impulse control, and negative impacts on attention and learning [10]. In addition, the aerosol produced by e-cigarettes contains cancer-causing chemicals and tiny particles that reach deep into the lungs [11].

Many young people want to quit vaping. Across social media platforms, posts, videos, tweets, and comments are ubiquitous from young people about the negative impact that vaping is having on their health and well-being and their desire to quit. Key themes include feeling addicted and unable to control their use of e-cigarettes; deleterious impacts on relationships with family, friends, and significant others; declining academic and athletic performance; concerns about job and career trajectories; and negative health experiences [12].

Unfortunately, the rapid increase of e-cigarette use among young people has left researchers, clinicians, and public health professionals without evidence to turn to about how to effectively support vaping cessation among young people, particularly at the scale necessary. A search for peer-reviewed manuscripts on e-cigarette cessation yields two case reports [13,14], both of which highlight the need for guidelines and research. The 2020 Surgeon General’s Report on Smoking Cessation [15] called for research to develop and understand safe and effective e-cigarette cessation interventions. Although vaping differs from smoking in many important ways, in the absence of scientific literature on vaping cessation, decades of research on best practices for smoking cessation likely provide a useful starting point for intervention design. Smoking cessation treatment delivered via text message has been shown to be effective among young adults [16-18], impacting key psychosocial processes that impact abstinence [19]. Mobile phone ownership is at 99% among young adults [20], and text messaging is an easy-to-use, discreet, anonymous, and preferred communication modality in this age group [21,22], making it a promising modality to reach and engage young people in vaping cessation treatment.

### Objectives

To our knowledge, this protocol describes the first-ever randomized controlled trial to evaluate the effectiveness of a vaping cessation intervention among young adults. The intervention is delivered entirely via text messages and is scalable at a national level. The primary aim of this study is to evaluate the effectiveness of a text message quit vaping program in promoting abstinence from e-cigarettes among young adults aged 18-24 years compared with an assessment-only control condition. Secondary aims are to examine changes in self-efficacy, perceived social norms, and social support for quitting as hypothesized mediators of program effectiveness and to examine if treatment effectiveness is moderated by gender, race/ethnicity, or sexual minority status. The primary hypothesis is that participants in the active intervention arm will be more likely to be abstinent at the 7-month postrandomization primary end point than participants in an assessment-only control arm.

## Methods

### Study Setting

The study is restricted to individuals in the United States. Recruitment, enrollment, and follow-up assessments are conducted via the web, and treatment is delivered via text messages. The study is conducted by Truth Initiative, and the study protocol was approved by the Advarra Institutional Review Board ([IRB] PRO00040067).

### Trial Design

This study is a 2-arm randomized controlled trial conducted among 2600 young adult e-cigarette users recruited through web advertisements for a study on vaping cessation. Participants will be randomized to the active text message intervention arm (n=1300) or to an assessment-only control arm (n=1300) in a 1:1 ratio following the methods described below.

### Inclusion Criteria

Individuals will be eligible if they are aged 18 to 24 years, own a mobile phone and have an active text message plan, are currently using e-cigarettes (defined as past 30-day use), are interested in quitting vaping in the next 30 days, and are a US resident.

### Exclusion Criteria

Individuals are excluded if they fail to provide contact information during the baseline assessment process (to ensure study retention), if they do not provide informed consent, or if they do not fully enroll in their assigned text message program by responding to the initial system-generated message.

### Recruitment and Enrollment

Web advertisements on various platforms (eg, Facebook and Twitter) will describe the study opportunity and lead to the study website, which provides details about study participation, including incentives for participation. Interested individuals will complete eligibility screening followed by informed consent and a baseline assessment. Those who complete the baseline will be randomized into 1 of the 2 arms and instructed to text a specific keyword to the phone number corresponding to their treatment assignment. Only those who respond to an initial opt-in confirmation message from the text message program within 24 hours will be fully enrolled into the study. This requirement will be made explicit.

### Informed Consent

Following eligibility screening, potential participants must provide informed consent to continue with the enrollment process. The web-based informed consent form provides details about the requirements for study participation, incentive structure, randomization process, plans for protecting human subjects data, and contact information for study staff and Advarra IRB. Agreeing to the informed consent will immediately launch the baseline assessment.

### Randomization

Randomization will occur at the completion of the baseline survey. A computer algorithm embedded in the survey software will automate random allocation in a 1:1 sequence. Investigators will be blind to treatment assignment.

### Interventions

Treatment attrition and loss to follow-up are particular challenges in digital interventions [23], especially among young people [24]. Differential attrition where follow-up rates are higher in one group than in another can bias results [25]. To minimize differential attrition and to optimize overall follow-up assessment completion rates, incentivized text messages asking about e-cigarette use and abstinence will be sent to all participants at 14 days post enrollment and then monthly thereafter through 6 months post randomization. At 14 days, enrollees will be asked, “Checking in: Have you cut down how much you vape nicotine in the past 2 weeks? Respond w/letter: A=I still use the same amount, B=I use less, C=I don’t use at all anymore.” At monthly intervals from 1 month post randomization to 6 months post randomization, enrollees will be asked, “How’s the quit going? When was the last time you vaped nicotine, even a puff of someone else’s? Respond w/ letter: A=in the past 7 days, B=8-30 days ago, C=More than 30 days ago.” Participants in both arms will be compensated US $5 via a digital gift card for each response to these text message assessments (total 7 assessments, possible payment US $35).

#### This is Quitting

In response to the youth vaping epidemic and the scarcity of available quitting resources for young people, in January 2019, Truth Initiative launched *This is Quitting*, a first-of-its-kind free e-cigarette cessation text message program designed specifically for young people [26]. *This is Quitting* is promoted nationally through *truth*, the public education campaign run by Truth Initiative for over 20 years [27], as well as through earned media and local and national outreach efforts. Since it was launched, more than 150,000 teens (aged 13-17 years) and young adults (aged 18-24 years) have enrolled. Observational data have shown that approximately 70% of enrolled users set a quit date, nearly half use one or more of the interactive keywords for on-demand support, and 68% stay enrolled for the full duration of the program. This study focuses on evaluating the effectiveness of the program among young adults.

*This is Quitting* is fully automated and interactive, grounded in best practices from smoking cessation research with young people [17,24,28] and our experience delivering digital tobacco cessation interventions to people of all ages, and informed by formative research with young e-cigarette users and quitters. The program is written in first-person, positioned as a nonjudgmental, supportive friend to the user. It is anchored around key constructs from the Social Cognitive Theory [29]. For example, to build self-efficacy, users receive messages that are designed to bolster confidence (eg, Matt says: “Just trust the process. It’s hard at first but it gets easier with time. And don’t get down on yourself, every day is a new day.” We all believe in you here*.*). To establish or reinforce perceived social norms and social support around quitting, a majority of messages come from other users who have submitted them to the program (edited by Truth Initiative personnel where appropriate). These messages reference the author and are designed to convey that many other young people are quitting and that the user is not alone (eg, Ashley says: “You can do it we are all in this together.” You’re not the only one who’s thought about quitting.). To support observational learning, users receive messages with strategies from other young people (eg, Dalton says: “Remember that stress can be dealt with in other ways! Try meditating or even writing down what the problem is and then figure out solutions.” You dealt with hard things before you started to vape, and you still can.) To grow behavioral capability, users receive messages that suggest concrete evidence-based skills and strategies users can practice (eg, Have your friends supported your quitting? Reply YES or NO. {{If user responds NO}} Practice - like actually say out loud in front of a mirror at home or in your car - how you’ll turn down a JUUL if they offer it to you.). Like this example, many of the messages are interactive in nature, encouraging users to engage with the program.

Participants typically receive 1 to 2 messages per day, with 3 messages sent on their quit date. Messages are tailored to users’ age, to their enrollment date or quit date (which can be set and reset via text message), and to the vape product they use (if provided by the user). Those not ready to quit receive 4 weeks of messages focused on building skills and confidence. Users who set a quit date receive messages for 1 week preceding it and 8 weeks afterward that include encouragement and support, skill- and self-efficacy building exercises, coping strategies, and information about the risks of vaping, benefits of quitting, and cutting down to quit. For young adult users, messages about nicotine replacement therapy state that it may make quitting more comfortable, that it is available over the counter, and that they should talk to a doctor or pharmacist to help determine the best dose. Keywords such as COPE, STRESS, SLIP, and MORE can be used to request on-demand support. Users can unsubscribe to stop receiving messages anytime by texting STOP. The message confirming unenrollment instructs them how to reengage with the program at any time and solicits feedback about the program.

To isolate the treatment benefit provided by the program from any confounding effects related to its public marketing or promotion, all branding and reference to the branded name will be removed from the program. Instead, it will be generically described as the *Quit Vaping Study* to participants randomized to this arm.

#### Assessment-Only Control

After an initial message confirming enrollment, participants will receive the incentivized text messages asking about e-cigarette use and abstinence, as described above. They will not receive any additional text messages. At the end of the intervention period and following the last follow-up assessment, they will receive information on how to sign up for *This is Quitting* (free and publicly available) if they are interested.

### Data Collection

The baseline survey will be conducted on the web and hosted on a secure server. Follow-up assessments at 1 month post randomization and 7 months post randomization will be conducted via mixed-mode follow-up. The 7-month postrandomization follow-up is designed to correspond to the 6-month posttreatment follow-up most commonly used in clinical trials and the end point used by quitlines in assessing the effectiveness of a real-world intervention where the end of treatment varies across participants [30]. Participants who do not complete the survey on the web will be contacted by phone and text message by the research staff. Telephone surveys will be conducted by research staff blind to treatment. Text messages will be used as a final means of gathering abstinence data from nonresponders.

There will be no payment for study enrollment or completion of the baseline assessment. Participants will be paid US $20 for completing each follow-up survey via the internet or over the phone with a telephone interviewer, with an additional US $10 incentive for responding within 24 hours of receiving the initial invitation.

### Measures

#### Screening Variables

To characterize those interested in the study, we will gather information about demographics (age, education, income level, sexual and gender identity, race, and ethnicity), current e-cigarette use (use of e-cigarettes containing nicotine or marijuana in the past 30 days [31]), and interest in quitting.

#### Baseline Variables

To characterize study participants and explore the potential moderators of treatment effectiveness, we will gather additional demographic information beyond the screener (student status and employment status); current e-cigarette use and history (frequency and rate [31,32], motivation to quit, and quitting history [33]); nicotine dependence will be assessed with the PROMISE-E [34] and items from the Texas Adolescent and Tobacco Marketing Surveillance [35]; other substance use (other tobacco products, alcohol [36]), and mental health symptoms [37]. Given the evolving trends of e-cigarette use and perception, we will also ask about their awareness of media reports about e-cigarettes, perception about e-cigarettes, reasons for wanting to quit, and reasons for joining the study. To account for potential predictors of treatment dropout, we will ask about motivation to use or quit e-cigarettes and potential barriers to quitting (eg, social influences).

#### Mediating Variables

The program primarily aims to help people quit vaping by building their self-confidence through skills training and by increasing perceived social norms and social support for quitting. Accordingly, we hypothesize that changes in self-efficacy and perceived social norms and social support will mediate the relationship between treatment assignment and abstinence outcomes. These constructs will be assessed at baseline, 1 month post randomization, and 7 months post randomization, and changes will be examined in mediational analyses. Items assess how supportive of quitting vaping their friends and family are, perceptions about how many of their peers vape or want to quit vaping, and awareness about media reports of vaping-related illnesses.

#### Primary Outcome

The primary outcome is self-reported 30-day abstinence at 7 months post randomization. Participants are first instructed “Please note that the terms ‘vape’ or ‘vaping’ in this survey refer to use of all vaping products, including JUUL, mods, and other e-cigarettes.” They are then asked to respond to the following question: “In the past 30 days, did you vape at all, even a puff of someone else’s?”

Following the completion of the final 7-month follow-up assessment, after participants receive compensation for completing this survey, we will administer an exit questionnaire that queries participants about the accuracy of their self-reports. This process was used successfully by Lantini et al [38] to determine rates of misreporting of outcomes in a randomized trial with adolescent smokers. Items will address the reasons for joining the study, the extent to which they paid attention to the study questions and their answers, the extent to which they like/dislike the way vaping makes them look, concerns about confidentiality in the study, and several items tapping social desirability bias in the context of a digital intervention.

#### Secondary Outcomes

In addition to the 7-month assessment of abstinence as the primary outcome, we will gather abstinence data at all other follow-ups as secondary measures. Other quitting-related outcomes include change in motivation to quit, quit attempts and quit methods, reduction in e-cigarette use, 7-day abstinence, and continuous abstinence measured at each formal follow-up as well as interim text message assessments (single items asking about current vaping status). Nicotine dependence among those still vaping will be assessed with the PROMISE-E [34] and items from the Texas Adolescent and Tobacco Marketing Surveillance [35]. We will ask about other substance use (other tobacco products and alcohol [36]) and mental health symptoms [37] as in the baseline survey. Intervention satisfaction in both conditions will be measured with items about overall satisfaction (1=very satisfied, 2=somewhat satisfied, 3=a little satisfied, and 4=not at all satisfied), how likely they would be to recommend the intervention to a friend (0=not at all likely and 10=very likely), and a rating of the number of text messages they received (1=too few, 2=just right, and 3=too many) [39]. To assess perceived message relevance, participants will be asked to provide feedback about the text messages by agreeing/disagreeing with several statements, such as if text messages “were written personally for me,” [40] “suggested quitting strategies that were new to me,” and “made me feel that I knew the right steps to take to quit.”

### Data Analysis Plan

#### Sample Generalizability

We will compare our final enrolled sample with those who did not complete the enrollment process to identify if study participants differ from the sample from which they were drawn [41]. These analyses will provide important information about the generalizability of our study sample to the broader population of young adult e-cigarette users interested in quitting vaping.

#### Pilot Analyses for Sample Size Calculations

Given that this is the first-ever evaluation of a quit vaping program, we were unable to draw on existing literature to estimate the potential effect size of our intervention against an assessment-only control to determine the appropriate sample size. Previous text message interventions for smoking cessation among young adults suggest a treatment benefit of 5 to 10 percentage points, favoring the intervention arm [42,43]. However, these studies vary by age of participants, abstinence metric, assessment end point, and obviously the nature of the behavior change being studied (smoking vs vaping). Therefore, we examined abstinence rates among an initial pilot sample of participants who completed the 1-month follow-up from our own trial to determine the final sample size. Details are provided below in the Results section.

#### Primary Outcome

Point prevalence abstinence at 7 months post randomization will be compared between the treatment and control groups using logistic regression. All estimates will be adjusted for baseline confounders of the intervention-outcome relationship. Missing data will be handled in 2 ways. First, we will conduct an intent-to-treat (ITT) analysis in which participants who have been lost to follow-up are assumed to be treatment failures (ie, vaping). This analysis will be conducted because ITT analyses are common in the smoking cessation literature, despite simulations demonstrating that the approach is neither conservative nor anticonservative but rather biased in favor of whichever condition contains less missingness [44]. Second, we will supplement ITT analyses with an analysis that uses a multiple imputation procedure to minimize bias in estimates and SEs, under the assumption that outcomes are not missing at random but rather more likely to be missing for treatment failures (ie, vaping) than treatment successes (ie, abstinence). Given that the magnitude of actual response bias is unknown, we will conduct a sensitivity analysis to evaluate the treatment effect on outcomes under a range of magnitudes, from equal odds of missing (odds ratio [OR] 1) to 5 times more likely to be missing (OR 5).

#### Secondary Outcomes

Additional outcomes related to abstinence and treatment engagement will also be analyzed with logistic regression as secondary analyses. These include the likelihood of making a quit attempt, likelihood of reducing e-cigarette use, and changes in confidence and self-efficacy in quitting e-cigarettes.

#### Moderators and Mediators of the Treatment-Outcome Effect

We will identify potential moderators (eg, age, gender, baseline motivation to quit) by analyzing interactions between treatment and selected variables. For all moderators found to be associated with the primary outcome, we will examine the effects of treatment/moderator interaction terms on outcomes after entering the main effects.

Our conceptual model is that treatment increases the odds of abstinence by increasing perceived social norms, perceived social support, and perceived self-efficacy for quitting. These 3 constructs will be measured at baseline, 1 month post randomization, and 7 months post randomization. Changes in these constructs from baseline will be evaluated with separate mediation analyses. Specifically, we hypothesize that the effect of treatment on abstinence will be mediated by changes in perceived social norms, perceived social support, and perceived self-efficacy, such that (1) a significant effect is found associating treatment assignment with changes in the mediator, (2) a significant effect is found associating changes in the mediator with outcome (abstinence), (3) a significant effect is found associating treatment with outcome (abstinence), and (4) the effect of the treatment-outcome relationship is significantly attenuated when the other effects (ie, 1 and 2) are simultaneously included in the model.

## Results

Study recruitment began on December 18, 2019, and is projected to be completed by spring 2020. The final 7-month follow-up is anticipated to be completed by fall/winter 2020.

Between December 18, 2019, and December 28, 2019, a total of 269 participants were randomized to treatment (This is Quitting: n=148 and control: n=121). The 1-month response rate was 79.2%, with no difference between arms. Using ITT analyses, 30-day abstinence rates were 16.2% (24/148) among those randomized to *This is Quitting* and 8.3% (10/121) among those randomized to control. A treatment difference of 16% vs 8% is detectable, with 80% power at 2-sided alpha=.05 with 260/group (520 total). To be able to detect treatment differences of this magnitude in a 20% subsample (eg, Hispanic or sexual minority young adult e-cigarette users), we determined that we needed to enroll 1300/group (2600 total) in our full sample.

## Discussion

### Significance and Challenges

This study is the first-ever comparative effectiveness trial of an intervention designed specifically to help young people quit vaping. It is fully powered to examine potentially important subgroup differences among young people who are more vulnerable to e-cigarette use. In addition, it lays the groundwork for future intervention research and begins to build an evidence base for vaping cessation treatment.

Selection of a control condition in a behavioral trial such as this one requires careful consideration and balance between internal and external validity [45]. Masking of treatment assignment can be difficult [46], and developing an *inactive* behavioral control arm that is credible and equally preferable to participants in the context of a text message intervention is a unique challenge. Participants enrolled in the trial seeking support to quit vaping and expected to receive some form of intervention. Therefore, we elected to use an assessment-only approach for the control arm to deliver an experience that retains subjects while not being so involved that it significantly changes participant behavior.

Conducting a comparative effectiveness trial of a quit vaping program during a time when the e-cigarette product [47-49] and policy [50-52] landscape are in flux is a unique challenge. It is important to acknowledge that myriad contextual factors from the individual level up through the policy level may influence the outcomes of this trial to a greater or lesser degree. We expect that whatever factors are at play and whatever degree of influence they may have, randomization should distribute such influence evenly across both arms.

### Limitations

Several aspects of our trial design and implementation are worth noting as potential limitations. First, the timing of funding availability meant that we launched the study several weeks before the new year. Recruiting during a time when motivation to quit may be higher than at other times during the year may result in somewhat inflated quit rates across the arms. Alternatively, it is possible that the motivation among individuals who attempt to quit vaping around the New Year is more ephemeral and characterized by less dedication and intensity, yielding perhaps lower quit rates than if the study was conducted at a different time of year. Previous research on smoking cessation has supported both possibilities. Regardless, we do not have any reason to believe that this seasonal influence would differentially affect participants in the 2 arms and that this potential confounding factor would be addressed via randomization.

Second, it is important to acknowledge that the active intervention arm is publicly available and being actively promoted through a national education campaign. Participants in both conditions may become aware of this national campaign at some point during the trial. Follow-up measures will assess awareness of the truth campaign and engagement in *This is Quitting* among control arm participants.

It is also important to note that we have no plans for biochemical verification of abstinence, although this is not necessarily a limitation given the context of this research. Previous digital cessation research has shown low response rates among young people despite a protocol involving minimal participant burden [53]. Indeed, biochemical confirmation is often not practical in large-scale trials with no in-person contact between participants and study staff and where the entirety of the study is conducted digitally [54,55], both of which characterize this trial. Furthermore, biomarkers are more useful for verifying brief periods of smoking abstinence than longer periods of abstinence (eg, our primary end point of 30-day abstinence). We believe that our measurement approach using the methods described by Lantini et al [38] will add important information about the veracity of self-reported cessation outcomes.

Finally, this study focuses on evaluating the effectiveness of the intervention only among young adults aged 18-24 years and does not include teens. Research among teens involves ethical and practical considerations (eg, parental assent) that are not present in research with adults aged 18 years and older. Given that the e-cigarette epidemic is largely concentrated among middle and high school students, it will be important to study this intervention among youth, with the appropriate ethical controls in place.

### Conclusions

Research on e-cigarettes, to date, has largely centered on their potential benefit as an alternative to cigarettes and their potential utility as a smoking cessation strategy [15]. While this often-contentious debate continues [56-58], there is an urgent and critical need to identify effective vaping cessation strategies to support the thousands—perhaps hundreds of thousands—of young people who want to quit vaping today. To our knowledge, this study is the first randomized controlled trial that is fully powered to evaluate the effectiveness of an automated, scalable, cost-efficient text message program for vaping cessation designed specifically for young people. Observational data from this program is extremely promising, both in terms of the massive uptake and engagement seen within the first year and also with respect to signals of abstinence. The data generated from this trial will establish a benchmark of effectiveness for other vaping cessation programs and begin to create a body of evidence focused on how best to help young people break free from e-cigarettes.

## Abbreviations

e-cigarette: electronic cigarette
IRB: Institutional Review Board
ITT: intent-to-treat
OR: odds ratio
